# NLRP3 Inflammasome: Potential Role in Obesity Related Low-Grade Inflammation and Insulin Resistance in Skeletal Muscle

**DOI:** 10.3390/ijms22063254

**Published:** 2021-03-23

**Authors:** Gonzalo Jorquera, Javier Russell, Matías Monsalves-Álvarez, Gonzalo Cruz, Denisse Valladares-Ide, Carla Basualto-Alarcón, Genaro Barrientos, Manuel Estrada, Paola Llanos

**Affiliations:** 1 Centro de Neurobiología y Fisiopatología Integrativa (CENFI), Facultad de Ciencias, Universidad de Valparaíso, Valparaíso 2360102, Chile; gonzalo.jorquera@uv.cl (G.J.); gonzalo.cruz@uv.cl (G.C.); 2 Escuela de Pedagogía en Educación Física, Facultad de Educación, Universidad Autónoma de Chile, Santiago 8900000, Chile; javier.russell@uautonoma.cl; 3 Instituto de Ciencias de la Salud, Universidad de O’Higgins, Rancagua 2820000, Chile; matias.monsalves@uoh.cl (M.M.-Á.); denisse.valladares@uoh.cl (D.V.-I.); 4 Departamento de Ciencias de la Salud, Universidad de Aysén, Coyhaique 5951537, Chile; carla.basualto@uaysen.cl; 5 Departamento de Anatomía y Medicina Legal, Facultad de Medicina, Universidad de Chile, Santiago 8380453, Chile; 6 Programa de Fisiología y Biofísica, ICBM, Facultad de Medicina, Universidad de Chile, Santiago 8380453, Chile; gbarrientos@med.uchile.cl (G.B.); iestrada@med.uchile.cl (M.E.); 7 Centro de Estudios en Ejercicio, Metabolismo y Cáncer, Facultad de Medicina, Universidad de Chile, Santiago 8380453, Chile; 8 Facultad de Odontología, Instituto de Investigación en Ciencias Odontológicas, Universidad de Chile, Santiago 8380544, Chile

**Keywords:** NALP3, chronic inflammation, muscle, glucose transport

## Abstract

Among multiple mechanisms, low-grade inflammation is critical for the development of insulin resistance as a feature of type 2 diabetes. The nucleotide-binding oligomerization domain-like receptor family (NOD-like) pyrin domain containing 3 (NLRP3) inflammasome has been linked to the development of insulin resistance in various tissues; however, its role in the development of insulin resistance in the skeletal muscle has not been explored in depth. Currently, there is limited evidence that supports the pathological role of NLRP3 inflammasome activation in glucose handling in the skeletal muscle of obese individuals. Here, we have centered our focus on insulin signaling in skeletal muscle, which is the main site of postprandial glucose disposal in humans. We discuss the current evidence showing that the NLRP3 inflammasome disturbs glucose homeostasis. We also review how NLRP3-associated interleukin and its gasdermin D-mediated efflux could affect insulin-dependent intracellular pathways. Finally, we address pharmacological NLRP3 inhibitors that may have a therapeutical use in obesity-related metabolic alterations.

## 1. Introduction

Metabolic disorders, such as obesity, insulin resistance (IR) and type 2 diabetes (T2D) are linked to a low-grade but chronic inflammatory state, also known as metabolic inflammation [1,2]. The precise pathways by which inflammation is triggered and maintained without an overt infection in these pathophysiological states are not fully understood. One current challenge is to find molecular sensors that can respond to environmental cues, such as the nutritional or metabolic status, which trigger the early phases of inflammatory cascades. Current evidence indicates the participation of the nucleotide-binding oligomerization domain-like receptor family (NOD-like) pyrin domain containing 3 (NLRP3) inflammasome in the development of inflammation and insulin resistance in diverse tissues [3,4,5,6,7], which are early stages in the pathogenesis of T2D. The NLRP3 inflammasome, which is the best-characterized inflammasome, has been implicated in the development of chronic diseases [4]. The mechanisms underlying the activation of NLRP3 inflammasome-dependent pathways are a current topic of great interest [8]. Here, we discuss how different stimuli, such as reactive oxygen species (ROS), ion flux and lysosomal destabilization, have crucial roles in inflammasome activation. We also present the available experimental evidence linking NLRP3 inflammasome activation with obesity in the skeletal muscle.

## 2. Skeletal Muscle as a Key Target Organ for Insulin Actions

**Figure 1:**
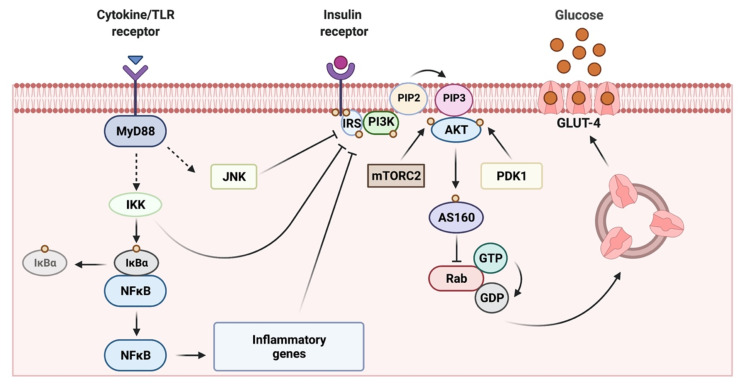
Crosstalk between inflammatory pathways and insulin-mediated glucose transport. Both Toll-like receptors (TLR) and cytokine receptor agonists—such as interleukin (IL)-1β—recruit the adaptor MyD88 (left). The activated MyD88, which involves the activation of the interleukin-1 receptor associated kinase (IRAK) family downstream, leads to the activation of the kinase inhibitor κ kinase (IKK), which in turn phosphorylates the nuclear factor kappa B (NF-κB) inhibitor IκBα, resulting in ubiquitin-dependent IκBα degradation. NF-κB is translocated to the nucleus and it initiates the transcription of proinflammatory genes. Furthermore, c-Jun N-terminal kinase (JNK) protein is activated through this pathway. JNK, IKK and inflammatory cytokines promote inhibitory phosphorylation in IRS on serine residues impacting insulin-mediated glucose transport (right). In physiological conditions, insulin binds to its receptor and recruits and phosphorylates IRS-1 on tyrosine residues, which then recruits PI3K via Src homology 2 (SH2) domains. PI3K promotes the phosphorylation of phosphatidylinositol bisphosphate (PIP2) at the plasma membrane to PIP3. Then, PIP3-dependent kinase (PDK) and mTORC2 phosphorylate and activate Akt. Activated Akt phosphorylates and inactivates AS160, allowing sustained Rabs activation of the trafficking of GLUT4 storage vesicles to the plasma membrane for the surface expression of GLUT4.

IR is defined as a process in which normal or elevated insulin levels produce a reduced biological response characterized by impaired sensitivity to insulin-mediated glucose disposal [9]. Skeletal muscle IR has been proposed as the primary defect leading to T2D [10,11,12]. According to recent data from the International Diabetes Federation, 415 million people are affected with T2D worldwide [13]. Moreover, it is projected that the number of patients with T2D will increase to 642 million people by 2040 [13]. In humans, skeletal muscle is the primary site for glucose uptake in the postprandial state [11]. Under euglycemic and hyperinsulinemic conditions, ~80% of glucose uptake occurs in skeletal muscle [12,14]. Insulin promotes glucose uptake into the muscle fibers by activating a complex cascade of phosphorylation–dephosphorylation pathways [9]. In skeletal muscle, insulin binds to the insulin receptor (InsR), leading to the phosphorylation of key tyrosine residues [15]. The phosphorylation of the InsR causes the migration of the insulin receptor substrate (IRS)-1 to the plasma membrane where it is phosphorylated. The phosphorylation of IRS-1 results in the activation of the p85 regulatory subunit of phosphatidylinositol (PI)-3 kinase (PI-3K) and the activation of its p110 catalytic subunit, which promotes an increase in phosphatidylinositol-3,4,5 triphosphate [15]. The activation of PI-3K results in the activation of Akt protein and the phosphorylation of Akt substrate 160 (AS160), which facilitates the translocation of glucose transporter type 4 (GLUT4)-containing vesicles from intracellular store compartments to the transverse tubules (TTs) network and sarcolemmal membrane, leading to subsequent glucose uptake [16]. In skeletal muscle fibers, the TTs are the major membrane surface, and they are the main site of insulin signaling, GLUT4 translocation and glucose uptake [17,18,19]. Under physiological conditions, the transported glucose is phosphorylated by hexokinase, which can be used in glycolysis to produce ATP or stored as glycogen [20]. Glucose can also be directed to the hexosamine or pentose pathways [21,22]. During IR states, both insulin-induced GLUT4 translocation and glucose uptake into the skeletal muscle are dramatically reduced [23,24]. An increased serine and threonine—instead of tyrosine—phosphorylation of IRS-1 has been shown to impair insulin signaling, leading to IR and T2D [25]. Interestingly, a crucial role of inflammatory cytokines has been proposed to mediate abnormal IRS-1 phosphorylation and other effects on the insulin signaling cascade [1,2] (Figure 1). 

## 3. Obesity-Related Chronic Low-Grade Inflammation in Skeletal Muscle and Its Contribution to Insulin Resistance

Obesity is usually associated with a chronic low-grade inflammatory state that is involved in the pathogenesis of IR, T2D, atherosclerosis and hepatic steatosis, among others [26]. The expression of several pro-inflammatory cytokines has been found to be elevated in adipose tissue, liver and skeletal muscle from obese and insulin-resistant animal models and human patients [26]. Inflammation and elevated proinflammatory cytokine levels, particularly in the skeletal muscle of obese individuals, have been associated with the expansion of muscle adipose deposits and immune cell infiltration [27,28,29]. In mice, one week of feeding with a high fat diet (HFD) caused a 76% increase in the presence of proinflammatory macrophages in skeletal muscle; after 10 weeks, macrophages remained elevated by 47% compared to normal control diet-fed mice [28]. Other studies have found that T cell infiltration in skeletal muscle was induced by HFD feeding and correlates with insulin resistance. T cells presented a proinflammatory TH1 phenotype, with increased signal transducer and activator of transcription 1 (STAT1) phosphorylation in skeletal muscle of obese mice [29]. In humans, markers for macrophages and T cells were upregulated in skeletal muscle from obese subjects compared to lean ones [29]. In T2D patients, inflammatory macrophage phenotype marker clusters were increased in skeletal muscle, which was associated to higher levels of both glycated hemoglobin (Hb1Ac) and fasting glycemia [30]. On the other hand, myocytes from obese T2D patients secreted higher levels of proinflammatory cytokines compared to non-obese non-diabetic individuals [31,32], suggesting a specific role of skeletal muscle fibers in the development of obesity-related inflammation. We have recently shown that isolated skeletal muscle fibers can express inflammatory markers in diet-induced obese mice, leading to muscle insulin resistance [33]. These data suggest that the contribution of muscle fibers to obesity-associated inflammation could complement immune cell infiltration being important factors for chronic low grade inflammation development.

The activation of intracellular inflammatory signaling in obese individuals is linked to stimuli such as high circulating levels of free fatty acids (FFA) and proinflammatory cytokines [34,35]. The activation of the enzymatic complex inhibitor κ kinase (IKK) leads to the phosphorylation of the nuclear factor κB inhibitor, IκBα, and its subsequent degradation through the proteasome [26]. This causes the translocation of nuclear factor κB (NF-κB) to the nucleus and promotes the gene expression of pro-inflammatory cytokines, chemokines and adhesion factors, among others [26]. The activation of c-Jun N-terminal kinase (JNK) and IKK, both serine kinases, disrupts insulin signaling and promotes insulin resistance through the phosphorylation of insulin receptor substrates, IRS-1 and IRS-2, in serine residues, causing their inactivation, in opposition to phosphorylation in tyrosine residues associated with insulin-dependent IRS-1 activation in healthy subjects [36]. The JNK inhibitory phosphorylation of IRS1/2 leads to a decreased recruitment of the PI-3K/Akt signaling pathway in response to insulin [37]. A JNK phosphorylation site on IRS-1 was mapped at serine-307, and it was shown that phosphorylation on this residue inhibited the interaction of IRS-1 with the InsR, as opposed to tyrosine phosphorylation of IRS-1, which promotes this protein interaction [38,39]. The inhibition of the PI3K/Akt pathway blocks almost all metabolic actions of insulin, including the stimulation of glucose transport, glycogen synthesis and lipid synthesis [35]. Thus, understanding the mechanisms that maintain the functionality of the InsR/IRS-1/PI-3K/Akt pathway, which directly impacts on GLUT4 translocation, is essential to develop new strategies to ameliorate IR and T2D.

Skeletal muscle represents ~40% of the body weight and constitutes the body’s largest organ in non-obese individuals [40]. Recent studies have shown that inflammation associated with obesity also occurs in skeletal muscle [1,31,32,33]; however, its cause and its role in IR and T2D development is not well understood. The following evidence supports the relevance of inflammation in skeletal muscle: (i) a broad range of experimental and clinical findings show that skeletal muscle acts as a secretory organ [40]; (ii) during physiological conditions (e.g., exercise), muscle fibers produce and secrete cytokines and other peptides—collectively classified as myokines—that may exert autocrine, paracrine and endocrine effects [40,41]. However, the current information on the involvement of these myokines during pathological conditions in skeletal muscle is limited; (iii) the list of myokines is growing and includes interleukins (IL) such as IL-1, IL-1Rα, IL-6, IL-8, IL-10, IL-15, tumor necrosis factor alpha (TNF-α) and monocyte chemoattractant protein (MCP-1) [26,34,40,42]. In addition, skeletal muscle expresses many of the innate immune system components, including biologically active cytokine receptors and Toll-like receptors (TLRs) [43]. For example, in obese individuals, high levels of saturated FFA can induce the phosphorylation and activation of JNK and IKK through TLR4 in adipocytes and skeletal muscle cells [44,45]. Besides, *TLR4* expression is upregulated in skeletal muscle from insulin-resistant and obese subjects [46]. Recently, it has been suggested that TLR4 mediates some of the metabolic effects of exercise on skeletal muscle [47]; however, its role in IR and T2D is far less explored. There is evidence showing that nutrients can directly activate inflammatory muscle response, possibly through TLR receptors. For example, parenteral administration of FFA to mice for 2 h induces JNK phosphorylation and IR in skeletal muscle and liver [48]. In the case of cytokine receptors, it has been proposed that HFD feeding stimulates the IL-1 type I receptor, mediating an inflammatory signaling cascade with NF-κB activation in the skeletal muscle of mice [49]. These relationships are summarized in Figure 1.

## 4. NLRP3 Inflammasome: Mechanisms of Activation and Downstream Effectors

**Figure 2:**
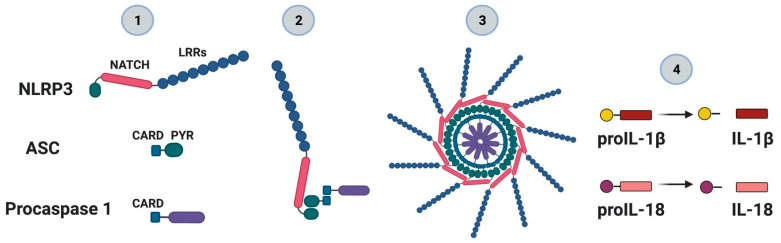
The nucleotide-binding oligomerization domain-like receptor family (NOD-like) pyrin domain containing 3 (NLRP3) inflammasome complex. The NLRP3 inflammasome is part of the NLR protein family which contains a pyridine 3 domain (NLRP3). The NLRP3 protein (1), through the internal interaction between the NACHT domain (or also referred to as NOD) and LRR (leucine-rich repeats), is self-repressed under normal cellular conditions. In the presence of pathogen-associated molecular patterns (PAMP) of microorganisms, damage-associated molecular patterns (DAMP) or metabolic perturbances, this self-repression is removed. The exposition of the NACHT domain leads to the oligomerization and recruitment of both ASC protein—through its PYR domain—and procaspase-1 through its CARD domain (2). Oligomerization or assembly triggers the activation of caspase-1 (3) and the processing of pro-inflammatory interleukins such as IL-1β and IL-18 (4).

**Figure 3:**
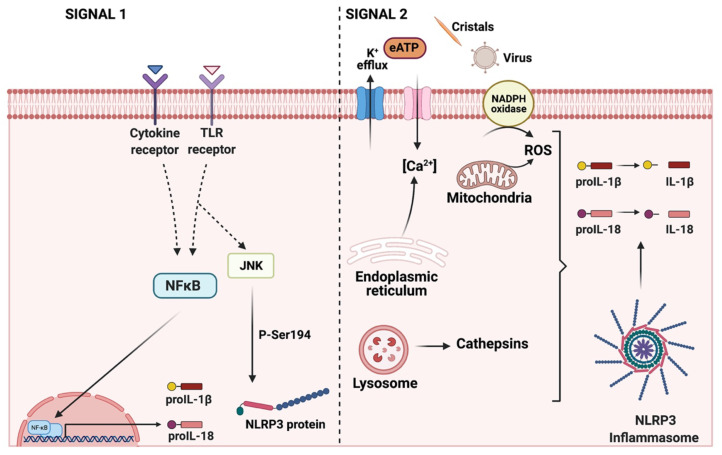
Activation of the NLRP3 inflammasome signaling pathway requires two signals. The signal 1 or priming (left) is provided by the activation of TLRs or cytokine receptors, leading to the NF-κB activation that upregulates the levels of several inflammasome components such as the protein NLRP3, proIL-1β and proIL-18. In addition, during the priming step, JNK-mediated phosphorylation occurs at S194 of NLRP3. Signal 2 or activation (right) is provided by any of numerous PAMPs or DAMPs including virus, cholesterol, potassium efflux, reactive oxygen species (ROS), extracellular ATP and lysosomal dysfunction, among others. ASC, an adaptor protein, recruits NLRP3 and procaspase-1 to form the NLRP3 inflammasome complex. Caspase 1 promotes the processing of interleukins for its subsequent release.

Metabolic inflammation is currently a very active research area, wherein aberrations in metabolic and inflammatory pathways contribute to IR and T2D [50]. Metabolic insults arising from obesity promote inflammation, which in turn can impair insulin signaling [51]. Since the discovery of the NLRP3 inflammasome, it has been suggested that this protein complex is a molecular link between metabolism and inflammation [52]. The NLRP3 inflammasome is formed through the interaction of a core of intracellular proteins identified as NLRP3 (for nucleotide-binding domain, leucine-rich containing family, pyrin domain-containing-3), bipartite adaptor protein ASC (an apoptosis-associated speck-like protein containing a caspase recruitment domain or CARD) and effector protein procaspase-1 [53]. The activated NLRP3 inflammasome cleaves procaspase-1 to its enzymatically activated form of caspase-1 and releases mature pro-inflammatory cytokines, such as IL-1β and IL-18 [50,54] (Figure 2). The maturation and release of IL-1β and IL-18 require two distinct steps (Figure 3): (i) the first step, known as priming, leads to the synthesis of pro-IL-1β and pro-IL-18 and other inflammasome components such as NLRP3 itself [8]. The priming signal, mediated by Toll-like receptors (TLR) activated by microbial components (called pathogen-associated molecular patterns, PAMPs) or by cytokine receptors activated by endogenous cytokines, promotes the NF-κB pathway to upregulate these proteins, the level of which is otherwise relatively low [8,55,56]. The activation of TLR signaling not only transcriptionally upregulates *NLRP3* expression but also post-transcriptionally activates NLRP3 by phosphorylation and deubiquitination [57]; (ii) the second step results in the assembly of the NLRP3 inflammasome, with caspase-1 activation and IL-1β and IL-18 secretion [50]. A variety of extracellular stimuli including extracellular ATP, pore-forming toxins, RNA viruses and particulate material are necessary for creating this second signal. In addition, multiple extracellular or intracellular events, including reactive oxygen species (ROS) generation, ionic flux and lysosomal damage, have also been suggested to activate the NLRP3 inflammasome [4,8,58] (Figure 3).

### 4.1. ROS-Mediated NLRP3 Inflammasome Activation

Obesity has been linked to increased ROS production, through mechanisms dependent on NADPH oxidase activity and mitochondrial dysfunction [59,60,61]. Exacerbated production of ROS has been reported to disrupt normal insulin response in skeletal muscle [62], leading to insulin resistance [63]. Interestingly, the inhibition of H_2_O_2_ producer enzyme—NADPH oxidase 2—by apocynin improves glucose tolerance and glucose uptake in the skeletal muscle of mice fed with an HFD [64]. In addition, a muscle-specific overexpression of catalase prevents the insulin resistance caused by an HFD through a decrease in mitochondrial ROS emission [65]. Despite these results, it is not clear which is the predominant cellular source of ROS during insulin resistance. Regarding the mechanism linking oxidative stress with insulin resistance, it has been described that the production of ROS promotes the activation of the serine/threonine kinases p38 MAPK, JNK and IKK, which are involved in the inhibitory phosphorylation of IRS-1 [62]. Increased ROS production has been proposed as a pivotal mechanism of NLRP3 inflammasome activation [58]. Thioredoxin-interacting protein (TXNIP) has been suggested as a link connecting the production of ROS and NLRP3 inflammasome activation [66]. TXNIP modulates the activity of the thioredoxin (TRX) redox system, thus influencing the cellular redox status in the cell. However, ROS production induces a dissociation of TXNIP/TRX, while it leads to an interaction between TXNIP/NLRP3 [66]. Evidence from different pathological conditions, including diabetic nephropathy [67], diabetic retinopathy [68], acute kidney injury [69], critical limb ischemia [70] or Alzheimer’s disease [71], have shown a relationship among oxidative stress, TXNIP and NLRP3. All of these results suggest that TXNIP is required for ROS-induced NLRP3 inflammasome activation. Interestingly, the expression of TXNIP in skeletal muscle is modulated by insulin, with a decrease in *TXNIP* mRNA levels observed in response to a euglycemic–hyperinsulinemic clamp [72]. These results suggest that insulin resistance could cause an alteration in the modulation of *TXNIP* expression. However, the precise molecular mechanism by which TXNIP induces NLRP3/ASC/pro-caspase 1 oligomerization and NLRP3 activation in skeletal muscle has not been determined yet. 

### 4.2. Ionic-Mediated Pathways for NLRP3 Inflammasome Activation

Early work showed that the NLRP3 complex is activated by nigericin [73,74] and maitotoxin [73]. Nigericin is an ionophore that induces both K^+^ efflux and H^+^ influx, leading to the activation of NLPR3 complex [75,76]. It has been suggested that obesity induces metabolic acidosis [77], which can acidify intracellular pH [78]. However, its role in the pathological activation of NLRP3 is not clear. 

The reported action mechanisms of maitotoxin are contradictory. It has been shown to form Na^+^ channel pores [79] and induce calcium entry in cultured cells [80] through the activation of verapamil-sensitive calcium channels [81]. Maitotoxin is the most potent marine toxin described to date [82] and activates TRPC1 at a picomolar concentration in Xenopus laevis oocytes [83]. TRCP1 is part of the store-operated calcium entry channels in many cell types and it forms non-selective cationic channels [84]. Therefore, it is likely that maitotoxin activates NLRP3 inflammasome through an intracellular calcium increase. A recent report shows that, in mice, obesity does not disturb the skeletal muscle calcium handling during contraction [85], suggesting that this calcium dysregulation could not be involved in NLRP3 activation in skeletal muscle. However, other calcium pathways might be involved in the activation of the skeletal muscle NLRP3 inflammasome. The activation of the extracellular calcium-sensing receptor pathway is engaged in inositol triphosphate (IP_3_) release and intracellular calcium signaling [86] and activates NLRP3 in monocytes [87]. Currently, the exact mechanism of NLRP3 activation by calcium is unknown. The inhibition of intracellular calcium increase mediated by endoplasmic reticulum, store-operated Ca^2+^ entry and Ca^2+^ entry from the extracellular milieu attenuate NLRP3 activation, and there is a consensus that intracellular Ca^2+^ increase is associated to K^+^ efflux [88]. Interestingly, glyburide, a sulfonylurea drug extensively used for the treatment of T2D, which inhibits the ATP-dependent potassium (K_ATP_) channel, blocking K^+^ efflux, has been identified as an NLRP3 inhibitor [89].

Skeletal muscle is an excitable tissue with tightly modulated intracellular ionic concentrations [90]. During muscle contraction, there is a significant K^+^ efflux, causing a 10–30 mM reduction in intracellular K^+^ concentration [91]. On the other hand, each contraction is promoted by a large intracellular calcium concentration increase [92]. Therefore, during the normal function of skeletal muscle, there is a significant K^+^ efflux and intracellular Ca^2+^ increase; however, it is unknown whether exercise activates NLRP3 through these processes. Thus, the activity of NLRP3 in skeletal muscle must be strongly regulated to prevent pathological activation during exercise. NLRP3 activation has been proposed to contribute to sarcopenia [93], and NLRP3 knockout is protected against inflammation-induced and Duchene associated sarcopenia [94,95]. However, the NLRP3 inflammasome activation mechanism in these pathologies remains undetermined.

In skeletal muscle, the stimulation with extracellular ATP (eATP) induces hypertrophy through IP3-induced intracellular calcium increase [96]. The sustained stimulation with mM levels of eATP induces the assembly of a large non-selective pore that permeates 900 Da molecules and is permeable to Na^+^ and Ca^2+^ and K^+^, disrupting cellular ionic homeostasis [97]. High levels of eATP activate intracellular kinases associated with inflammation, triggering insulin resistance in hepatocytes and adipocytes [98,99]. Recently, it has been shown that eATP is elevated in the skeletal muscle of HFD-fed mice, and eATP is responsible for triggering an inflammatory response—with IL-1β upregulation—and insulin resistance in skeletal muscle [33]. New studies are necessary to evaluate whether elevated levels of eATP promote metabolic disturbances in skeletal muscle cells from obese individuals through ionic flux alteration and NLRP3 activation.

### 4.3. Lysosomal Dysfunction-Mediated NLRP3 Inflammasome Activation

Recently, it has been demonstrated that there is a significant lysosomal dysregulation in the adipose tissue of obese mice. For example, in the white adipose tissue (WAT) of HFD-induced obese mice and in ob/ob mice, the lysosomal cysteine protease activity of cathepsin L was decreased compared to control animals [100]. Some authors also found an accumulation of autophagosomes in WAT adipocytes from obese mice, suggesting that lysosomal dysfunction could contribute to autophagic alterations [100,101], which can promote the upregulation of IL-1β, IL-6 and MCP-1 in adipose cells [102]. In the hepatocytes of obese mice, lysosomes present a reduced acidification, causing a reduction in the lysosomal proteolytic activity of cathepsins B and L [103]. It has been proposed that these lysosomal alterations are triggered by an excess of intracellular lipids content in hepatic cells [101]. In skeletal muscle under physiological conditions, the lysosomal–autophagic pathway degrades a large amount of damaged proteins, attenuating the oxidative damage within muscle cells [104]. Interestingly, a decrease in the expression of cathepsin L has been found in the skeletal muscle of HFD-fed mice [105]. On the other hand, in control C2C12 muscle cells, the use of chloroquine, a lysosomal inhibitor, reduced insulin-mediated Akt phosphorylation and insulin sensitivity [106]. Several factors that disrupt the function and homeostasis of lysosomes activate NLRP3. Among these factors, inorganic crystals [107], the disruption of lysosomes associated to K^+^ efflux [55,108] and the accumulation of autophagosomes and lysosomes [109] have been described. Nonetheless, the molecular pathways that connect lysosomal dysregulation and NLRP3 activation are unclear. In endothelial cells, the damage induced by palmitate can be prevented by the use of simvastatin, which improves lysosome function and consequently reduces NLRP3 inflammasome activation [110]. The authors proposed that simvastatin promotes lysosome and autophagosome biogenesis, reducing lysosome injury and the release of lysosomal cathepsins and mediating the blockage of NLRP3-dependent cell damage [110]. On the other hand, some cathepsins have been associated with NLRP3 activation. In a renal ischemia/reperfusion injury model, the downregulation of cathepsin B and L reduce NLRP3 function and NF-κB signaling [111]. Besides, obese human subjects show consistent elevations in cathepsin S levels in blood [112]. Accordingly, extracellular cathepsin S has been proposed as a biomarker for lysosomal disruption [113], which might be associated with NLRP3 activation. In line with this, knockout mice for cathepsin S fed with an HFD showed lower levels of blood glucose compared to HFD-fed WT mice, plus a reduction in hepatic glucose production [114]. However, in skeletal muscle, there is still no evidence of the role of lysosomal cathepsins in the activation of the NLRP3 inflammasome in an obesogenic context.

## 5. Gasdermins: A Specific Pathway for IL-1β Secretion

NLRP3 inflammasome activity promotes the maturation and subsequent release of IL-1β and IL-18 through caspase-1-mediated cleavage from their inactive precursors [50,54]. Members of the IL-1 family lack a signal peptide related to protein processing and secretion, thus impeding IL-1β from being secreted by the classic exocytic pathway [115]. Alternative routes have been proposed for the secretion of these cytokines; a new family of proteins known as Gasdermins (GSDMs) have been shown to accomplish this role [115,116]. GSDMs are characterized by their ability to form pores in the plasma membrane [117]. Upon GSDM caspase mediated-cleavage and the association between their N-terminal ends, the complex can be inserted in the plasma membrane, leading to the formation of pores that lack ion selectivity [117]. Six GSDM proteins have been described to date in humans: GSDMA, GSDMB, GSDMC, GSDMD, GSDME (DFNA5) and GSDMF (DFNB59) [118,119]. GDSMD is the only member of the family that harbors an inflammatory caspase cleavage site, thus rendering the active protein in an inflammatory context [118]. The basal GSDMD conformation maintains the protein in an autoinhibitory state. After activation, the GSDMD N-terminal domains oligomerize to generate membrane pores (of around 10–15 nm diameter), which could be the route for IL-1β secretion, since it was demonstrated that primary bone-marrow-derived macrophage knockout for GSDMD significantly decreased their IL-1β secretion [120]. These and other observations led investigators to conclude that GSDMD was necessary for IL-1β release and that GSDMD pores were not associated with cell death in monocytes [118]. Furthermore, it has been described that IL-1β can be secreted by hyperactive phagocytes (viable cells), as opposed to dying cells [116]. 

The majority of the functions for the different GSDM family members have been described in leukocytes, upper gastrointestinal tract and liver [116,121,122], but for skeletal muscle, information is very limited. We have observed increased GSDMD protein expression in muscle fibers from HFD-fed mice compared to controls (Llanos P, unpublished results). In a recent publication, Hu et al. (2020) inhibited GSDMD-mediated cytokine release by using Disulfiram (DSF) in human monocytic THP-1 cell line, mouse immortalized bone marrow-derived macrophages, and also in septic mice models [119]. By modifying the residue Cys191 that only exists in GSDMD, DSF blocks pore formation [119]. Relating GSDM inhibition and obesity, a recent work demonstrated that using DSF in the diet of HFD-fed mice for 64 weeks prevented the metabolic alterations observed in untreated HFD-fed mice. DSF was shown to improve fasting blood glucose levels, induce a loss in adiposity, and a decrease in body weight in obese animals [123]. Although this study did not deepen our understanding of the molecular mechanisms behind these positive effects, the anti-obesity properties of this drug offer interesting ways to study GSDMD function in highly metabolic tissues affected by obesity, as in the case of skeletal muscle. 

## 6. NLRP3 Role in Metabolic Disorders: Limited Information in Skeletal Muscle

The aberrant activation of the NLRP3 inflammasome is associated with the pathogenesis of various inflammatory, autoimmune and metabolic diseases, including atherosclerosis and T2D [124,125]. In line with this concept, mice genetically deficient in NLRP3 are protected against HFD–induced insulin resistance [126,127]. The NLRP3 inflammasome pathway has been well characterized in cells participating in innate immunity [128]; however, there is limited information regarding its expression and activation in non-hematopoietic cells, such as skeletal muscle fibers. Nonetheless, there is evidence showing that the NLRP3 inflammasome appears to be involved in myopathies [129], muscle atrophy [94] and sarcopenia [93].

Interestingly, *NRLP3* mRNA expression in humans is higher in skeletal muscle biopsies from subjects that eat a high palmitate diet than those eating a low-palmitate/high-oleate diet [130]. The insulin-induced phosphorylation of Akt in the skeletal muscle of *NLRP3*^−/−^ HFD-fed mice was similar or potentiated compared to their littermate controls [5,6], suggesting that the NLRP3 inflammasome can mediate obesity-associated deleterious signals in the skeletal muscle and contribute to obesity-induced inflammation and IR [5]. In concordance, the IRS-1/Akt cascade can be restored by pharmacological inhibition of NLRP3 activity in both the liver and skeletal muscle of HFD-fed mice [127]. However, the effect of NLRP3 inhibition has been evaluated mainly in basal conditions, and its effect in an insulin-stimulated context remains poorly explored. Additionally, in an animal model of dementia (PLB2_TAU_), glucose homeostasis is altered, displaying inflammation and glucose intolerance [131]. The pharmacological inhibition of NLRP3 improved glycemia management in PLB2_TAU_ animals, with an increment in InsR (Tyr1162/1163) phosphorylation, increased IRS1 protein levels and reduced JNK phosphorylation levels in the skeletal muscle and liver, showing that NLRP3 could have an active role in the metabolic signaling of insulin target organs [131]. All these data suggest a link between NLRP3 inflammasome and insulin signaling; however, relevant aspects of this NLRP3 metabolic role such as its influence over glucose uptake and GLUT4 translocation have not been studied in skeletal muscle. 

In obese and T2D patients, there is an elevation of multiple cytokines, including those with NLRP3-dependent activation, such as IL-1β and IL-18 [132,133,134]. Both IL-1β and IL-18 are members of the IL-1 family [135]. Less is known about IL-1β in skeletal muscle compared with other tissues with a high metabolic rate. In vitro studies have shown that chronic treatment with IL-1β slightly decreases *GLUT4* expression and markedly inhibits its insulin-induced translocation to the plasma membrane in murine and human adipocytes [136,137]. It has been reported that HFD feeding stimulates IL-1 type I receptor (IL-1R), mediating intracellular inflammatory signaling in murine skeletal muscle, suggesting that the IL-1R type I/Myeloid differentiation primary response 88 (MyD88)/NF-κB signaling pathway is involved in the skeletal muscle inflammatory response in a diet-induced obesity model [49]. Notably, the NF-κB pathway controls the expression of inflammatory cytokines involved in the pathogenesis of insulin resistance [31,138]. Elevated NF-κB activation is conserved in human myocytes cultured from obese T2D patients, confirming the muscle’s contribution to the inflammatory response [31]. Moreover, hypercholesterolemia induces inflammation by the activation of TLR-dependent pathways and, subsequently, the NF-κB-mediated release of a broad range of cytokines and chemokines, including IL-1β [139]. 

There is limited evidence regarding the cellular mechanisms engaging IL-18 signaling pathways. Human skeletal muscle expresses *IL-18* mRNA, but a role for IL-18 in muscle remains elusive. A plasma infusion of TNF-α in human subjects increases *IL-18* expression in muscle and reduces insulin-mediated glucose uptake [140]. The authors propose that TNF-α and IL-18 may interact, and both could have critical regulatory roles in the pathogenesis of insulin resistance [140]. However, a more recent work showed that IL-18 knockout mice develop spontaneous obesity due to lipid accumulation. Moreover, when IL-18 null mice were exposed to HFD, the ensuing obesity phenotype was exacerbated [141]. These controversial findings indicate that more research is needed to clarify the role of IL-18 in obesity and IR.

## 7. Skeletal Muscle Lipid Infiltration: Possible Role on Local Inflammation?

Skeletal muscle has a crucial role in whole-body metabolism, including glucose uptake, the amino acid reservoir and fatty acid oxidation [48,142]. In obesity conditions, skeletal muscle is infiltrated with lipids, causing a reduction in its fatty acid β-oxidation and altering its insulin sensitivity [143,144]. In skeletal muscle, intramyocellular lipids (IMCLs) are stored as triglyceride-rich lipid droplets depending on the muscle fiber type, with a higher content in type I fibers compared to type II [145,146]. Lipid increments in intermuscular deposits and the saturation with IMCL have been shown to alter glucose homeostasis independent of age [147]. Interestingly, this IMCL accretion seems to be detrimental in obesity and T2D but not for endurance-trained athletes, who exhibit high oxidative capacity and enhanced insulin sensitivity despite their high levels of lipids within skeletal muscle, a process known as the “athlete paradox” [148,149]. However, more recently, both the location (such as sarcolemmal, cytosolic, mitochondrial/ER) and lipid composition are likely to be relevant for insulin action impairments [150,151,152,153]. In obesity, the parallel increase of lipid intermediates (e.g., diacylglycerol, sphingolipids, acylcarnitine and long-chain acyl-CoA) promotes the lipotoxic potential and lipid role in inflammation development [154]. For example, ceramide, a sphingosine-based intracellular lipid increased by cytokines such as TNF-α and IL-1β, can block protein synthesis in vitro [155]. Actually, it has been proposed that obesity—and particularly the degree of inflammation—has a powerful impact on the disruption of anabolic signaling [156,157]. Furthermore, skeletal muscle myoblasts treated in vitro with palmitate show ceramide accumulation, increased *forkhead box O3* (FoXO3) and *MaFbx* mRNA levels and impaired protein synthesis, resulting in muscle atrophy [158]. In this context, in *NLRP3*^−/−^ C2C12 cells, atrophy is reduced when myotubes were exposed to an inflammatory insult [94]. On the other hand, it has been shown that perilipin PLIN2, one of five lipid droplet-associated proteins responsible for triglyceride storage, can alter insulin-mediated glucose uptake by the activation of NLRP3 and subsequent IL-1β increase on C2C12 cells [159].

Regarding the role of NLPR3 in the adipose tissue of obese mice, the pharmacological inhibition of the NLRP3 inflammasome has been demonstrated to improve lipid and glucose handling, reducing cytokine secretion, fibrosis and adipose tissue remodeling, suggesting a potential therapeutic role for metabolic alterations associated to obesity [160]. Meanwhile, in skeletal muscle, our understanding of NLRP3 is still limited, and whether muscle tissue releases pro-inflammatory cytokines induced by increased IMCL or if the NLRP3 mediates this effect is currently unknown. It has been stated that, in mice and human skeletal muscle, an HFD induces an expansion of intramuscular lipids and IMCL, and these increments are associated with T-cell infiltration and differentiation to a pro-inflammatory Th1 phenotype with exacerbated STAT1 phosphorylation [29]. Lipid-induced local secretion of MCP-1 is associated with the expression of inflammatory markers such as IL-1β, TNFα, and the recruitment of macrophages within the skeletal muscle of obese mice and T2D patients [161]. These results imply that a lipotoxic environment induced by either HFD or a diabetic state can promote leukocytes recruitment to skeletal muscle. However, it is unknown if this leukocyte recruitment is mediated by IMCL expansion associated with obesity and T2D. The current available data suggest a relevant role for lipid infiltration over the inflammatory signaling that could account for skeletal muscle acting as an autocrine/paracrine organ to potentiate local low-grade chronic inflammation, as seen in adipose tissue; however, its potential participationover NRLP3 inflammasome activation in skeletal muscle needs further investigation.

## 8. NLRP3 Inflammasome Inhibitors

Several drugs can inhibit NLRP3 but with nonspecific effects on other targets. In 2015, MCC950—a diarylsulfonylurea-containing compound—was presented as a specific inhibitor of NLRP3, leading to impaired IL-1β processing and decreased secretion of the cytokine [54]. The authors also showed that MCC950 does not inhibit other members of the inflammasome family such as NLRC4 and NLRP1, both of which are involved in the response to microbial infections [54]. There is no evidence evaluating the NLRP3 inflammasome blockade with MCC950 in skeletal muscle as a therapeutic approach for obese IR individuals. However, several pieces of evidence show improvements in different inflammatory conditions. For example, attenuating the NLRP3 inflammasome using MCC950 reduces myocardial fibrosis after coronary artery ligation in mice [162]. Besides this, in diabetic mice, the vascular neointima hyperplasia induced by hyperglycemia associated with NLRP3 activation is inhibited with MCC950 [163]. Finally, MCC950 in vivo treatment has been shown to reduce inflammation and muscle damage in a valosin-containing protein myopathy mice model [129]. T2D is associated with cognitive impairments and neuropsychiatric abnormalities named diabetic encephalopathy, with an inflammatory component in its pathogenesis [164]. In diabetic db/db mice, treatment with MCC950 improves insulin sensitivity and attenfuates anxiety and depression-like behaviors and cognitive dysfunctions [165]. Interestingly, the hormone fibroblast growth factor 21 (FGF21) mimics the effect of MCC950 by suppressing NLRP3 activation through spleen tyrosine kinase (Syk) phosphorylation, thus representing a potential alternative to inhibit the NLRP3 inflammasome [163]. Syk inhibition with the selective inhibitor EMD638683 also has anti-inflammatory effects due to inhibiting NLRP3 inflammasome activation, preventing angiotensin II-induced cardiac inflammation and fibrosis [164]. 

Another interesting NLRP3 inflammasome inhibitor is β-hydroxybutyrate (βHB) [166,167]. Ketone bodies are released to blood circulation by the liver during fasting, carbohydrate restriction and prolonged exercise [166]. βHB has been proposed as a signaling molecule that can endogenously inhibit class I histone deacetylases, leading to hyperacetylation and modulating gene expression, altering in particular the activity of the stress response FoXO3 transcription factor [168]. As an NLRP3 inflammasome inhibitor, βHB, through the HCAR2 receptor, has a role in preventing retinal damage in diabetic db/db mice [166]. Furthermore, some reports show a beneficial effect against brain [167] and liver [168] inflammatory damage mediated by NLRP3; however, whether βHB has an impact on NLRP3 inflammasome activation in skeletal muscle is currently unknown. Thomsen et al. (2018) showed that the infusion of βHB resulted in an attenuation of muscle amino acid degradation under an LPS infusion protocol in human subjects [169], suggesting that, in the presence of acute inflammation, which is a known condition in which the NLRP3 inflammasome is activated [8], βHB could act as an anti-inflammatory and anticatabolic molecule, with positive effects on skeletal muscle health and function; however, its association with NLRP3 remains to be elucidated.

## 9. Conclusions and Perspectives

**Figure 4:**
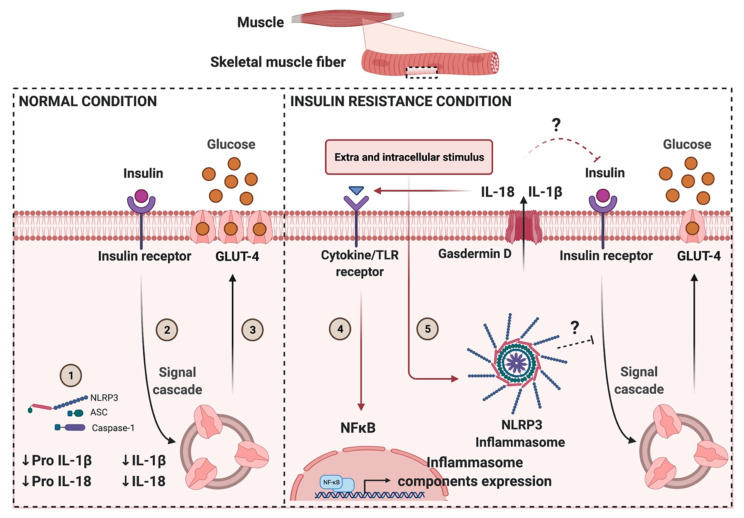
Schematic figure showing NLRP3 inflammasome activation and its possible modulation on insulin-mediated signaling in skeletal muscle during normal and insulin resistance conditions. In normal conditions, the expression of the NLRP3 inflammasome components is low, suggesting that the priming signal is inhibited. Consequently, there is a decreased expression and processing of pro-interleukins (1). In response to insulin (2), the GLUT4 transporters translocate to the sarcolemma and T-tubule system, promoting glucose transport (3). During IR conditions, an increased priming signal promotes the NF-κB mediated expression of the NLRP3 inflammasome components, plus their assembly and activation. Several extracellular and intracellular stimuli may play a role in NLRP3 inflammasome activation, highlighting the importance of free fatty acids (FFA), ionic flux, extracellular ATP (eATP) and ROS, among others, which could lead to the proteolytic activation of the pro-inflammatory cytokines IL-1β and IL-18 and its Gasdermins D (GSDMD)-mediated secretion (4,5). Excessive or prolonged NLRP3-dependent pro-inflammatory cytokine exposure may cause IR in skeletal muscle fibers.

Obesity and IR are associated with the reduced insulin-mediated translocation of GLUT4 in skeletal muscle which directly impairs glucose transport (Figure 1). Mounting evidence suggests that in IR, there is a first signal that increases the expression of the NLRP3 inflammasome complex components in skeletal muscle and a second signal involved in their assembly, which in turn increases the maturation and release of pro-inflammatory cytokines. Little is known about how the skeletal muscle NLRP3 inflammasome might disrupt normal insulin signaling. How the activation of the NLRP3 inflammasome impairs glucose homeostasis at the muscle level is an open question. We have presented a significant amount of evidence that suggests that cytokines processed by the NLRP3 inflammasome and potentially released by skeletal muscle could have an autocrine or paracrine inhibitory effect on insulin signaling, leading to insulin resistance (Figure 4). Unraveling these mechanisms may open new therapeutic targets for treating this pervasive metabolic condition.

## References

[1] Wu H., Ballantyne C.M. (2017). Skeletal muscle inflammation and insulin resistance in obesity. J. Clin. Investig..

[2] McArdle M.A., Finucane O.M., Connaughton R.M., McMorrow A.M., Roche H.M. (2013). Mechanisms of obesity-induced inflammation and insulin resistance: Insights into the emerging role of nutritional strategies. Front. Endocrinol..

[3] Stienstra R., van Diepen J.A., Tack C.J., Zaki M.H., van de Veerdonk F.L., Perera D., Neale G.A., Hooiveld G.J., Hijmans A., Vroegrijk I. (2011). Inflammasome is a central player in the induction of obesity and insulin resistance. Proc. Natl. Acad. Sci. USA.

[4] Ozaki E., Campbell M., Doyle S.L. (2015). Targeting the NLRP3 inflammasome in chronic inflammatory diseases: Current perspectives. J. Inflamm. Res..

[5] Vandanmagsar B., Youm Y.H., Ravussin A., Galgani J.E., Stadler K., Mynatt R.L., Ravussin E., Stephens J.M., Dixit V.D. (2011). The NLRP3 inflammasome instigates obesity-induced inflammation and insulin resistance. Nat. Med..

[6] Wen H., Gris D., Lei Y., Jha S., Zhang L., Huang M.T., Brickey W.J., Ting J.P. (2011). Fatty acid-induced NLRP3-ASC inflammasome activation interferes with insulin signaling. Nat. Immunol..

[7] Stienstra R., Joosten L.A., Koenen T., van Tits B., van Diepen J.A., van den Berg S.A., Rensen P.C., Voshol P.J., Fantuzzi G., Hijmans A. (2010). The inflammasome-mediated caspase-1 activation controls adipocyte differentiation and insulin sensitivity. Cell Metab..

[8] Jo E.K., Kim J.K., Shin D.M., Sasakawa C. (2016). Molecular mechanisms regulating NLRP3 inflammasome activation. Cell Mol. Immunol..

[9] Wilcox G. (2005). Insulin and insulin resistance. Clin. Biochem. Rev..

[10] Ferrannini E., Smith J.D., Cobelli C., Toffolo G., Pilo A., DeFronzo R.A. (1985). Effect of insulin on the distribution and disposition of glucose in man. J. Clin. Investig..

[11] DeFronzo R.A., Gunnarsson R., Björkman O., Olsson M., Wahren J. (1985). Effects of insulin on peripheral and splanchnic glucose metabolism in noninsulin-dependent (type II) diabetes mellitus. J. Clin. Investig..

[12] DeFronzo R.A., Tripathy D. (2009). Skeletal muscle insulin resistance is the primary defect in type 2 diabetes. Diabetes Care.

[13] Ogurtsova K., da Rocha Fernandes J.D., Huang Y., Linnenkamp U., Guariguata L., Cho N.H., Cavan D., Shaw J.E., Makaroff L.E. (2017). IDF Diabetes Atlas: Global estimates for the prevalence of diabetes for 2015 and 2040. Diabetes Res. Clin. Pract..

[14] Thiebaud D., Jacot E., DeFronzo R.A., Maeder E., Jequier E., Felber J.P. (1982). The effect of graded doses of insulin on total glucose uptake, glucose oxidation, and glucose storage in man. Diabetes.

[15] Klip A., Sun Y., Chiu T.T., Foley K.P. (2014). Signal transduction meets vesicle traffic: The software and hardware of GLUT4 translocation. Am. J. Physiol. Cell Physiol..

[16] Karlsson H.K., Zierath J.R., Kane S., Krook A., Lienhard G.E., Wallberg-Henriksson H. (2005). Insulin-stimulated phosphorylation of the Akt substrate AS160 is impaired in skeletal muscle of type 2 diabetic subjects. Diabetes.

[17] Lauritzen H.P., Ploug T., Prats C., Tavaré J.M., Galbo H. (2006). Imaging of insulin signaling in skeletal muscle of living mice shows major role of T-tubules. Diabetes.

[18] Wang W., Hansen P.A., Marshall B.A., Holloszy J.O., Mueckler M. (1996). Insulin unmasks a COOH-terminal Glut4 epitope and increases glucose transport across T-tubules in skeletal muscle. J. Cell Biol..

[19] Lauritzen H.P., Galbo H., Brandauer J., Goodyear L.J., Ploug T. (2008). Large GLUT4 vesicles are stationary while locally and reversibly depleted during transient insulin stimulation of skeletal muscle of living mice: Imaging analysis of GLUT4-enhanced green fluorescent protein vesicle dynamics. Diabetes.

[20] Rose A.J., Richter E.A. (2005). Skeletal muscle glucose uptake during exercise: How is it regulated?. Physiology.

[21] Love D.C., Hanover J.A. (2005). The hexosamine signaling pathway: Deciphering the “O-GlcNAc code”. Sci. Stke.

[22] Wagner K.R., Kauffman F.C., Max S.R. (1978). The pentose phosphate pathway in regenerating skeletal muscle. Biochem. J..

[23] Garvey W.T., Maianu L., Zhu J.H., Brechtel-Hook G., Wallace P., Baron A.D. (1998). Evidence for defects in the trafficking and translocation of GLUT4 glucose transporters in skeletal muscle as a cause of human insulin resistance. J. Clin. Investig..

[24] Zierath J.R., He L., Gumà A., Odegoard Wahlström E., Klip A., Wallberg-Henriksson H. (1996). Insulin action on glucose transport and plasma membrane GLUT4 content in skeletal muscle from patients with NIDDM. Diabetologia.

[25] Hotamisligil G.S., Peraldi P., Budavari A., Ellis R., White M.F., Spiegelman B.M. (1996). IRS-1-mediated inhibition of insulin receptor tyrosine kinase activity in TNF-alpha- and obesity-induced insulin resistance. Science.

[26] Gregor M.F., Hotamisligil G.S. (2011). Inflammatory mechanisms in obesity. Annu. Rev. Immunol..

[27] Sinacore D.R., Gulve E.A. (1993). The role of skeletal muscle in glucose transport, glucose homeostasis, and insulin resistance: Implications for physical therapy. Phys. Ther..

[28] Fink L.N., Costford S.R., Lee Y.S., Jensen T.E., Bilan P.J., Oberbach A., Blüher M., Olefsky J.M., Sams A., Klip A. (2014). Pro-inflammatory macrophages increase in skeletal muscle of high fat-fed mice and correlate with metabolic risk markers in humans. Obesity.

[29] Khan I.M., Perrard X.Y., Brunner G., Lui H., Sparks L.M., Smith S.R., Wang X., Shi Z.Z., Lewis D.E., Wu H. (2015). Intermuscular and perimuscular fat expansion in obesity correlates with skeletal muscle T cell and macrophage infiltration and insulin resistance. Int. J. Obes..

[30] Fink L.N., Oberbach A., Costford S.R., Chan K.L., Sams A., Blüher M., Klip A. (2013). Expression of anti-inflammatory macrophage genes within skeletal muscle correlates with insulin sensitivity in human obesity and type 2 diabetes. Diabetologia.

[31] Green C.J., Pedersen M., Pedersen B.K., Scheele C. (2011). Elevated NF-κB activation is conserved in human myocytes cultured from obese type 2 diabetic patients and attenuated by AMP-activated protein kinase. Diabetes.

[32] Ciaraldi T.P., Ryan A.J., Mudaliar S.R., Henry R.R. (2016). Altered Myokine Secretion Is an Intrinsic Property of Skeletal Muscle in Type 2 Diabetes. PLoS ONE.

[33] Jorquera G., Meneses-Valdés R., Rosales-Soto G., Valladares-Ide D., Campos C., Silva-Monasterio M., Llanos P., Cruz G., Jaimovich E., Casas M. (2021). High extracellular ATP levels released through pannexin-1 channels mediate inflammation and insulin resistance in skeletal muscle fibers of diet induced obese mice. Diabetologia.

[34] Ye J. (2013). Mechanisms of insulin resistance in obesity. Front. Med..

[35] Nandipati K.C., Subramanian S., Agrawal D.K. (2017). Protein kinases: Mechanisms and downstream targets in inflammation-mediated obesity and insulin resistance. Mol. Cell Biochem..

[36] Hirosumi J., Tuncman G., Chang L., Görgün C.Z., Uysal K.T., Maeda K., Karin M., Hotamisligil G.S. (2002). A central role for JNK in obesity and insulin resistance. Nature.

[37] Sabio G., Kennedy N.J., Cavanagh-Kyros J., Jung D.Y., Ko H.J., Ong H., Barrett T., Kim J.K., Davis R.J. (2010). Role of muscle c-Jun NH2-terminal kinase 1 in obesity-induced insulin resistance. Mol. Cell Biol..

[38] Aguirre V., Uchida T., Yenush L., Davis R., White M.F. (2000). The c-Jun NH(2)-terminal kinase promotes insulin resistance during association with insulin receptor substrate-1 and phosphorylation of Ser(307). J. Biol. Chem..

[39] Aguirre V., Werner E.D., Giraud J., Lee Y.H., Shoelson S.E., White M.F. (2002). Phosphorylation of Ser307 in insulin receptor substrate-1 blocks interactions with the insulin receptor and inhibits insulin action. J. Biol. Chem..

[40] Pedersen B.K., Febbraio M.A. (2012). Muscles, exercise and obesity: Skeletal muscle as a secretory organ. Nat. Rev. Endocrinol..

[41] Pedersen B.K. (2013). Muscle as a secretory organ. Compr. Physiol..

[42] Solinas G., Karin M. (2010). JNK1 and IKKbeta: Molecular links between obesity and metabolic dysfunction. FASEB J..

[43] Lang C.H., Silvis C., Deshpande N., Nystrom G., Frost R.A. (2003). Endotoxin stimulates in vivo expression of inflammatory cytokines tumor necrosis factor alpha, interleukin-1beta, -6, and high-mobility-group protein-1 in skeletal muscle. Shock.

[44] Shi H., Kokoeva M.V., Inouye K., Tzameli I., Yin H., Flier J.S. (2006). TLR4 links innate immunity and fatty acid-induced insulin resistance. J. Clin. Investig..

[45] Martins A.R., Nachbar R.T., Gorjao R., Vinolo M.A., Festuccia W.T., Lambertucci R.H., Cury-Boaventura M.F., Silveira L.R., Curi R., Hirabara S.M. (2012). Mechanisms underlying skeletal muscle insulin resistance induced by fatty acids: Importance of the mitochondrial function. Lipids Health Dis..

[46] Reyna S.M., Ghosh S., Tantiwong P., Meka C.S., Eagan P., Jenkinson C.P., Cersosimo E., Defronzo R.A., Coletta D.K., Sriwijitkamol A. (2008). Elevated toll-like receptor 4 expression and signaling in muscle from insulin-resistant subjects. Diabetes.

[47] Ali M.M., McMillan R.P., Fausnacht D.W., Kavanaugh J.W., Harvey M.M., Stevens J.R., Wu Y., Mynatt R.L., Hulver M.W. (2020). Muscle-specific Deletion of Toll-like Receptor 4 Impairs Metabolic Adaptation to Wheel Running in Mice. Med. Sci. Sports Exerc..

[48] Tancredi R.G., Dagenais G.R., Zierler K.L. (1976). Free fatty acid metabolism in the forearm at rest: Muscle uptake and adipose tissue release of free fatty acids. Johns Hopkins Med. J..

[49] Kim S.J., Choi Y., Jun H.S., Kim B.M., Na H.K., Surh Y.J., Park T. (2010). High-fat diet stimulates IL-1 type I receptor-mediated inflammatory signaling in the skeletal muscle of mice. Mol. Nutr. Food Res..

[50] Duewell P., Kono H., Rayner K.J., Sirois C.M., Vladimer G., Bauernfeind F.G., Abela G.S., Franchi L., Nuñez G., Schnurr M. (2010). NLRP3 inflammasomes are required for atherogenesis and activated by cholesterol crystals. Nature.

[51] Kirwan A.M., Lenighan Y.M., O’Reilly M.E., McGillicuddy F.C., Roche H.M. (2017). Nutritional modulation of metabolic inflammation. Biochem. Soc. Trans..

[52] Haneklaus M., O’Neill L.A. (2015). NLRP3 at the interface of metabolism and inflammation. Immunol. Rev..

[53] Grant R.W., Dixit V.D. (2013). Mechanisms of disease: Inflammasome activation and the development of type 2 diabetes. Front. Immunol..

[54] Coll R.C., Robertson A.A., Chae J.J., Higgins S.C., Muñoz-Planillo R., Inserra M.C., Vetter I., Dungan L.S., Monks B.G., Stutz A. (2015). A small-molecule inhibitor of the NLRP3 inflammasome for the treatment of inflammatory diseases. Nat. Med..

[55] Hornung V., Latz E. (2010). Critical functions of priming and lysosomal damage for NLRP3 activation. Eur. J. Immunol..

[56] Guarda G., Zenger M., Yazdi A.S., Schroder K., Ferrero I., Menu P., Tardivel A., Mattmann C., Tschopp J. (2011). Differential expression of NLRP3 among hematopoietic cells. J. Immunol..

[57] Song N., Li T. (2018). Regulation of NLRP3 Inflammasome by Phosphorylation. Front. Immunol..

[58] Tschopp J., Schroder K. (2010). NLRP3 inflammasome activation: The convergence of multiple signalling pathways on ROS production?. Nat. Rev. Immunol..

[59] Marseglia L., Manti S., D’Angelo G., Nicotera A., Parisi E., Di Rosa G., Gitto E., Arrigo T. (2014). Oxidative stress in obesity: A critical component in human diseases. Int. J. Mol. Sci..

[60] McMurray F., Patten D.A., Harper M.E. (2016). Reactive Oxygen Species and Oxidative Stress in Obesity-Recent Findings and Empirical Approaches. Obesity.

[61] Diaz-Vegas A., Sanchez-Aguilera P., Krycer J.R., Morales P.E., Monsalves-Alvarez M., Cifuentes M., Rothermel B.A., Lavandero S. (2020). Is Mitochondrial Dysfunction a Common Root of Noncommunicable Chronic Diseases?. Endocr. Rev..

[62] Henriksen E.J., Diamond-Stanic M.K., Marchionne E.M. (2011). Oxidative stress and the etiology of insulin resistance and type 2 diabetes. Free Radic. Biol. Med..

[63] Di Meo S., Iossa S., Venditti P. (2017). Skeletal muscle insulin resistance: Role of mitochondria and other ROS sources. J. Endocrinol..

[64] Espinosa A., Campos C., Díaz-Vegas A., Galgani J.E., Juretic N., Osorio-Fuentealba C., Bucarey J.L., Tapia G., Valenzuela R., Contreras-Ferrat A. (2013). Insulin-dependent H2O2 production is higher in muscle fibers of mice fed with a high-fat diet. Int. J. Mol. Sci..

[65] Anderson E.J., Lustig M.E., Boyle K.E., Woodlief T.L., Kane D.A., Lin C.T., Price J.W., Kang L., Rabinovitch P.S., Szeto H.H. (2009). Mitochondrial H2O2 emission and cellular redox state link excess fat intake to insulin resistance in both rodents and humans. J. Clin. Investig..

[66] Zhou R., Tardivel A., Thorens B., Choi I., Tschopp J. (2010). Thioredoxin-interacting protein links oxidative stress to inflammasome activation. Nat. Immunol..

[67] Han Y., Xu X., Tang C., Gao P., Chen X., Xiong X., Yang M., Yang S., Zhu X., Yuan S. (2018). Reactive oxygen species promote tubular injury in diabetic nephropathy: The role of the mitochondrial ros-txnip-nlrp3 biological axis. Redox. Biol..

[68] Chen W., Zhao M., Zhao S., Lu Q., Ni L., Zou C., Lu L., Xu X., Guan H., Zheng Z. (2017). Activation of the TXNIP/NLRP3 inflammasome pathway contributes to inflammation in diabetic retinopathy: A novel inhibitory effect of minocycline. Inflamm. Res..

[69] Wen Y., Liu Y.R., Tang T.T., Pan M.M., Xu S.C., Ma K.L., Lv L.L., Liu H., Liu B.C. (2018). mROS-TXNIP axis activates NLRP3 inflammasome to mediate renal injury during ischemic AKI. Int. J. Biochem. Cell Biol..

[70] Elshaer S.L., Mohamed I.N., Coucha M., Altantawi S., Eldahshan W., Bartasi M.L., Shanab A.Y., Lorys R., El-Remessy A.B. (2017). Deletion of TXNIP Mitigates High-Fat Diet-Impaired Angiogenesis and Prevents Inflammation in a Mouse Model of Critical Limb Ischemia. Antioxidants.

[71] Li L., Ismael S., Nasoohi S., Sakata K., Liao F.F., McDonald M.P., Ishrat T. (2019). Thioredoxin-Interacting Protein (TXNIP) Associated NLRP3 Inflammasome Activation in Human Alzheimer’s Disease Brain. J. Alzheimer’s Dis..

[72] Parikh H., Carlsson E., Chutkow W.A., Johansson L.E., Storgaard H., Poulsen P., Saxena R., Ladd C., Schulze P.C., Mazzini M.J. (2007). TXNIP regulates peripheral glucose metabolism in humans. PLoS Med..

[73] Mariathasan S., Weiss D.S., Newton K., McBride J., O’Rourke K., Roose-Girma M., Lee W.P., Weinrauch Y., Monack D.M., Dixit V.M. (2006). Cryopyrin activates the inflammasome in response to toxins and ATP. Nature.

[74] Schilling W.P., Wasylyna T., Dubyak G.R., Humphreys B.D., Sinkins W.G. (1999). Maitotoxin and P2Z/P2X(7) purinergic receptor stimulation activate a common cytolytic pore. Am. J. Physiol..

[75] Margolis L.B., Rozovskaja I.A., Skulachev V.P. (1987). Acidification of the interior of Ehrlich ascites tumor cells by nigericin inhibits DNA synthesis. FEBS Lett..

[76] Pressman B.C. (1976). Biological applications of ionophores. Annu. Rev. Biochem..

[77] Adeva M.M., Souto G. (2011). Diet-induced metabolic acidosis. Clin. Nutr..

[78] Salameh A.I., Ruffin V.A., Boron W.F. (2014). Effects of metabolic acidosis on intracellular pH responses in multiple cell types. Am. J. Physiol. Regul Integr. Comp. Physiol..

[79] Nishio M., Muramatsu I., Yasumoto T. (1996). Na(+)-permeable channels induced by maitotoxin in guinea-pig single ventricular cells. Eur. J. Pharm..

[80] Murata M., Gusovsky F., Yasumoto T., Daly J.W. (1992). Selective stimulation of Ca^2+^ flux in cells by maitotoxin. Eur. J. Pharm..

[81] Ohizumi Y., Kajiwara A., Yasumoto T. (1983). Excitatory effect of the most potent marine toxin, maitotoxin, on the guinea-pig vas deferens. J. Pharm. Exp. Ther..

[82] Holmes M.J., Lewis R.J. (1994). Purification and characterisation of large and small maitotoxins from cultured Gambierdiscus toxicus. Nat. Toxins.

[83] Flores P.L., Rodríguez E., Zapata E., Carbó R., Farías J.M., Martínez M. (2017). Maitotoxin Is a Potential Selective Activator of the Endogenous Transient Receptor Potential Canonical Type 1 Channel in Xenopus laevis Oocytes. Mar. Drugs.

[84] Skopin A., Shalygin A., Vigont V., Zimina O., Glushankova L., Mozhayeva G.N., Kaznacheyeva E. (2013). TRPC1 protein forms only one type of native store-operated channels in HEK293 cells. Biochimie.

[85] Jaque-Fernandez F., Beaulant A., Berthier C., Monteiro L., Allard B., Casas M., Rieusset J., Jacquemond V. (2020). Preserved Ca. Diabetologia.

[86] Hannan F.M., Kallay E., Chang W., Brandi M.L., Thakker R.V. (2018). The calcium-sensing receptor in physiology and in calcitropic and noncalcitropic diseases. Nat. Rev. Endocrinol..

[87] Jäger E., Murthy S., Schmidt C., Hahn M., Strobel S., Peters A., Stäubert C., Sungur P., Venus T., Geisler M. (2020). Calcium-sensing receptor-mediated NLRP3 inflammasome response to calciprotein particles drives inflammation in rheumatoid arthritis. Nat. Commun..

[88] Elliott E.I., Sutterwala F.S. (2015). Initiation and perpetuation of NLRP3 inflammasome activation and assembly. Immunol Rev..

[89] Lamkanfi M., Mueller J.L., Vitari A.C., Misaghi S., Fedorova A., Deshayes K., Lee W.P., Hoffman H.M., Dixit V.M. (2009). Glyburide inhibits the Cryopyrin/Nalp3 inflammasome. J. Cell Biol..

[90] Mukund K., Subramaniam S. (2020). Skeletal muscle: A review of molecular structure and function, in health and disease. Wiley Interdiscip. Rev. Syst. Biol. Med..

[91] Sejersted O.M., Sjøgaard G. (2000). Dynamics and consequences of potassium shifts in skeletal muscle and heart during exercise. Physiol. Rev..

[92] Marcucci L., Canato M., Protasi F., Stienen G.J.M., Reggiani C. (2018). A 3D diffusional-compartmental model of the calcium dynamics in cytosol, sarcoplasmic reticulum and mitochondria of murine skeletal muscle fibers. PLoS ONE.

[93] McBride M.J., Foley K.P., D’Souza D.M., Li Y.E., Lau T.C., Hawke T.J., Schertzer J.D. (2017). The NLRP3 inflammasome contributes to sarcopenia and lower muscle glycolytic potential in old mice. Am. J. Physiol. Endocrinol. Metab..

[94] Huang N., Kny M., Riediger F., Busch K., Schmidt S., Luft F.C., Slevogt H., Fielitz J. (2017). Deletion of Nlrp3 protects from inflammation-induced skeletal muscle atrophy. Intensive Care Med. Exp..

[95] Boursereau R., Abou-Samra M., Lecompte S., Noel L., Brichard S.M. (2018). Downregulation of the NLRP3 inflammasome by adiponectin rescues Duchenne muscular dystrophy. BMC Biol..

[96] Ito N., Ruegg U.T., Takeda S. (2018). ATP-Induced Increase in Intracellular Calcium Levels and Subsequent Activation of mTOR as Regulators of Skeletal Muscle Hypertrophy. Int. J. Mol. Sci..

[97] Coutinho-Silva R., Persechini P.M. (1997). P2Z purinoceptor-associated pores induced by extracellular ATP in macrophages and J774 cells. Am. J. Physiol..

[98] Enjyoji K., Kotani K., Thukral C., Blumel B., Sun X., Wu Y., Imai M., Friedman D., Csizmadia E., Bleibel W. (2008). Deletion of cd39/entpd1 results in hepatic insulin resistance. Diabetes.

[99] Yu Z., Jin T. (2010). Extracellular high dosages of adenosine triphosphate induce inflammatory response and insulin resistance in rat adipocytes. Biochem. Biophys. Res. Commun..

[100] Mizunoe Y., Sudo Y., Okita N., Hiraoka H., Mikami K., Narahara T., Negishi A., Yoshida M., Higashibata R., Watanabe S. (2017). Involvement of lysosomal dysfunction in autophagosome accumulation and early pathologies in adipose tissue of obese mice. Autophagy.

[101] Mizunoe Y., Kobayashi M., Tagawa R., Nakagawa Y., Shimano H., Higami Y. (2019). Association between Lysosomal Dysfunction and Obesity-Related Pathology: A Key Knowledge to Prevent Metabolic Syndrome. Int. J. Mol. Sci..

[102] Yoshizaki T., Kusunoki C., Kondo M., Yasuda M., Kume S., Morino K., Sekine O., Ugi S., Uzu T., Nishio Y. (2012). Autophagy regulates inflammation in adipocytes. Biochem. Biophys. Res. Commun..

[103] Inami Y., Yamashina S., Izumi K., Ueno T., Tanida I., Ikejima K., Watanabe S. (2011). Hepatic steatosis inhibits autophagic proteolysis via impairment of autophagosomal acidification and cathepsin expression. Biochem. Biophys. Res. Commun..

[104] Kim Y., Triolo M., Hood D.A. (2017). Impact of Aging and Exercise on Mitochondrial Quality Control in Skeletal Muscle. Oxid. Med. Cell Longev..

[105] Huang X., Vaag A., Carlsson E., Hansson M., Ahrén B., Groop L. (2003). Impaired cathepsin L gene expression in skeletal muscle is associated with type 2 diabetes. Diabetes.

[106] Chang Y.C., Liu H.W., Chen Y.T., Chen Y.A., Chen Y.J., Chang S.J. (2018). Resveratrol protects muscle cells against palmitate-induced cellular senescence and insulin resistance through ameliorating autophagic flux. J. Food Drug Anal..

[107] Hornung V., Bauernfeind F., Halle A., Samstad E.O., Kono H., Rock K.L., Fitzgerald K.A., Latz E. (2008). Silica crystals and aluminum salts activate the NALP3 inflammasome through phagosomal destabilization. Nat. Immunol..

[108] Katsnelson M.A., Lozada-Soto K.M., Russo H.M., Miller B.A., Dubyak G.R. (2016). NLRP3 inflammasome signaling is activated by low-level lysosome disruption but inhibited by extensive lysosome disruption: Roles for K+ efflux and Ca^2+^ influx. Am. J. Physiol. Cell Physiol..

[109] Lauterbach M.A., Saavedra V., Mangan M.S.J., Penno A., Thiele C., Latz E., Kuerschner L. (2020). 1-Deoxysphingolipids cause autophagosome and lysosome accumulation and trigger NLRP3 inflammasome activation. Autophagy.

[110] Zhang Y., Wang Y.T., Koka S., Hussain T., Li X. (2020). Simvastatin improves lysosome function via enhancing lysosome biogenesis in endothelial cells. Front. Biosci..

[111] Tang T.T., Lv L.L., Pan M.M., Wen Y., Wang B., Li Z.L., Wu M., Wang F.M., Crowley S.D., Liu B.C. (2018). Hydroxychloroquine attenuates renal ischemia/reperfusion injury by inhibiting cathepsin mediated NLRP3 inflammasome activation. Cell Death Dis..

[112] Naour N., Rouault C., Fellahi S., Lavoie M.E., Poitou C., Keophiphath M., Eberlé D., Shoelson S., Rizkalla S., Bastard J.P. (2010). Cathepsins in human obesity: Changes in energy balance predominantly affect cathepsin s in adipose tissue and in circulation. J. Clin. Endocrinol. Metab..

[113] Hughes C.S., Colhoun L.M., Bains B.K., Kilgour J.D., Burden R.E., Burrows J.F., Lavelle E.C., Gilmore B.F., Scott C.J. (2016). Extracellular cathepsin S and intracellular caspase 1 activation are surrogate biomarkers of particulate-induced lysosomal disruption in macrophages. Part. Fibre Toxicol..

[114] Lafarge J.C., Pini M., Pelloux V., Orasanu G., Hartmann G., Venteclef N., Sulpice T., Shi G.P., Clément K., Guerre-Millo M. (2014). Cathepsin S inhibition lowers blood glucose levels in mice. Diabetologia.

[115] Kuriakose T., Kanneganti T.D. (2018). Gasdermin D Flashes an Exit Signal for IL-1. Immunity.

[116] Evavold C.L., Ruan J., Tan Y., Xia S., Wu H., Kagan J.C. (2018). The Pore-Forming Protein Gasdermin D Regulates Interleukin-1 Secretion from Living Macrophages. Immunity.

[117] Ramos-Junior E.S., Morandini A.C. (2017). Gasdermin: A new player to the inflammasome game. Biomed. J..

[118] Shi J., Gao W., Shao F. (2017). Pyroptosis: Gasdermin-Mediated Programmed Necrotic Cell Death. Trends Biochem. Sci..

[119] Hu J.J., Liu X., Xia S., Zhang Z., Zhang Y., Zhao J., Ruan J., Luo X., Lou X., Bai Y. (2020). FDA-approved disulfiram inhibits pyroptosis by blocking gasdermin D pore formation. Nat. Immunol..

[120] Shi J., Zhao Y., Wang K., Shi X., Wang Y., Huang H., Zhuang Y., Cai T., Wang F., Shao F. (2015). Cleavage of GSDMD by inflammatory caspases determines pyroptotic cell death. Nature.

[121] Saeki N., Kuwahara Y., Sasaki H., Satoh H., Shiroishi T. (2000). Gasdermin (Gsdm) localizing to mouse Chromosome 11 is predominantly expressed in upper gastrointestinal tract but significantly suppressed in human gastric cancer cells. Mamm. Genome..

[122] Khanova E., Wu R., Wang W., Yan R., Chen Y., French S.W., Llorente C., Pan S.Q., Yang Q., Li Y. (2018). Pyroptosis by caspase11/4-gasdermin-D pathway in alcoholic hepatitis in mice and patients. Hepatology.

[123] Bernier M., Mitchell S.J., Wahl D., Diaz A., Singh A., Seo W., Wang M., Ali A., Kaiser T., Price N.L. (2020). Disulfiram Treatment Normalizes Body Weight in Obese Mice. Cell Metab..

[124] Davis B.K., Wen H., Ting J.P. (2011). The inflammasome NLRs in immunity, inflammation, and associated diseases. Annu. Rev. Immunol..

[125] Guo H., Callaway J.B., Ting J.P. (2015). Inflammasomes: Mechanism of action, role in disease, and therapeutics. Nat. Med..

[126] Collino M., Benetti E., Rogazzo M., Mastrocola R., Yaqoob M.M., Aragno M., Thiemermann C., Fantozzi R. (2013). Reversal of the deleterious effects of chronic dietary HFCS-55 intake by PPAR-δ agonism correlates with impaired NLRP3 inflammasome activation. Biochem. Pharm..

[127] Chiazza F., Couturier-Maillard A., Benetti E., Mastrocola R., Nigro D., Cutrin J.C., Serpe L., Aragno M., Fantozzi R., Ryffel B. (2016). Targeting the NLRP3 Inflammasome to Reduce Diet-Induced Metabolic Abnormalities in Mice. Mol. Med..

[128] Martinon F., Tschopp J. (2004). Inflammatory caspases: Linking an intracellular innate immune system to autoinflammatory diseases. Cell.

[129] Nalbandian A., Khan A.A., Srivastava R., Llewellyn K.J., Tan B., Shukr N., Fazli Y., Kimonis V.E., BenMohamed L. (2017). Activation of the NLRP3 Inflammasome Is Associated with Valosin-Containing Protein Myopathy. Inflammation.

[130] Kien C.L., Bunn J.Y., Fukagawa N.K., Anathy V., Matthews D.E., Crain K.I., Ebenstein D.B., Tarleton E.K., Pratley R.E., Poynter M.E. (2015). Lipidomic evidence that lowering the typical dietary palmitate to oleate ratio in humans decreases the leukocyte production of proinflammatory cytokines and muscle expression of redox-sensitive genes. J. Nutr. Biochem..

[131] Hull C., Dekeryte R., Buchanan H., Kamli-Salino S., Robertson A., Delibegovic M., Platt B. (2020). NLRP3 inflammasome inhibition with MCC950 improves insulin sensitivity and inflammation in a mouse model of frontotemporal dementia. Neuropharmacology.

[132] Dinarello C.A., Wolff S.M. (1993). The role of interleukin-1 in disease. N. Engl. J. Med..

[133] Pickup J.C., Chusney G.D., Thomas S.M., Burt D. (2000). Plasma interleukin-6, tumour necrosis factor alpha and blood cytokine production in type 2 diabetes. Life Sci..

[134] Esposito K., Marfella R., Giugliano D. (2004). Plasma interleukin-18 concentrations are elevated in type 2 diabetes. Diabetes Care.

[135] Okamura H., Tsutsi H., Komatsu T., Yutsudo M., Hakura A., Tanimoto T., Torigoe K., Okura T., Nukada Y., Hattori K. (1995). Cloning of a new cytokine that induces IFN-gamma production by T cells. Nature.

[136] Jager J., Grémeaux T., Cormont M., Le Marchand-Brustel Y., Tanti J.F. (2007). Interleukin-1beta-induced insulin resistance in adipocytes through down-regulation of insulin receptor substrate-1 expression. Endocrinology.

[137] Lagathu C., Yvan-Charvet L., Bastard J.P., Maachi M., Quignard-Boulangé A., Capeau J., Caron M. (2006). Long-term treatment with interleukin-1beta induces insulin resistance in murine and human adipocytes. Diabetologia.

[138] Carlsen H., Haugen F., Zadelaar S., Kleemann R., Kooistra T., Drevon C.A., Blomhoff R. (2009). Diet-induced obesity increases NF-kappaB signaling in reporter mice. Genes Nutr..

[139] Tall A.R., Yvan-Charvet L. (2015). Cholesterol, inflammation and innate immunity. Nat. Rev. Immunol..

[140] Krogh-Madsen R., Plomgaard P., Møller K., Mittendorfer B., Pedersen B.K. (2006). Influence of TNF-alpha and IL-6 infusions on insulin sensitivity and expression of IL-18 in humans. Am. J. Physiol. Endocrinol. Metab..

[141] Murphy A.J., Kraakman M.J., Kammoun H.L., Dragoljevic D., Lee M.K., Lawlor K.E., Wentworth J.M., Vasanthakumar A., Gerlic M., Whitehead L.W. (2016). IL-18 Production from the NLRP1 Inflammasome Prevents Obesity and Metabolic Syndrome. Cell Metab..

[142] Jensen M.D., Ekberg K., Landau B.R. (2001). Lipid metabolism during fasting. Am. J. Physiol. Endocrinol. Metab..

[143] Kelley D.E., Goodpaster B., Wing R.R., Simoneau J.A. (1999). Skeletal muscle fatty acid metabolism in association with insulin resistance, obesity, and weight loss. Am. J. Physiol..

[144] Kim J.Y., Hickner R.C., Cortright R.L., Dohm G.L., Houmard J.A. (2000). Lipid oxidation is reduced in obese human skeletal muscle. Am. J. Physiol. Endocrinol. Metab..

[145] Daemen S., Gemmink A., Brouwers B., Meex R.C.R., Huntjens P.R., Schaart G., Moonen-Kornips E., Jörgensen J., Hoeks J., Schrauwen P. (2018). Distinct lipid droplet characteristics and distribution unmask the apparent contradiction of the athlete’s paradox. Mol. Metab..

[146] van Loon L.J. (2004). Use of intramuscular triacylglycerol as a substrate source during exercise in humans. J. Appl. Physiol..

[147] Kim J.E., Dunville K., Li J., Cheng J.X., Conley T.B., Couture C.S., Campbell W.W. (2017). Intermuscular Adipose Tissue Content and Intramyocellular Lipid Fatty Acid Saturation Are Associated with Glucose Homeostasis in Middle-Aged and Older Adults. Endocrinol. Metab..

[148] Goodpaster B.H., He J., Watkins S., Kelley D.E. (2001). Skeletal muscle lipid content and insulin resistance: Evidence for a paradox in endurance-trained athletes. J. Clin. Endocrinol. Metab..

[149] Dubé J.J., Amati F., Stefanovic-Racic M., Toledo F.G., Sauers S.E., Goodpaster B.H. (2008). Exercise-induced alterations in intramyocellular lipids and insulin resistance: The athlete’s paradox revisited. Am. J. Physiol. Endocrinol. Metab..

[150] Samjoo I.A., Safdar A., Hamadeh M.J., Glover A.W., Mocellin N.J., Santana J., Little J.P., Steinberg G.R., Raha S., Tarnopolsky M.A. (2013). Markers of skeletal muscle mitochondrial function and lipid accumulation are moderately associated with the homeostasis model assessment index of insulin resistance in obese men. PLoS ONE.

[151] Nielsen J., Mogensen M., Vind B.F., Sahlin K., Højlund K., Schrøder H.D., Ortenblad N. (2010). Increased subsarcolemmal lipids in type 2 diabetes: Effect of training on localization of lipids, mitochondria, and glycogen in sedentary human skeletal muscle. Am. J. Physiol. Endocrinol. Metab..

[152] Bergman B.C., Goodpaster B.H. (2020). Exercise and Muscle Lipid Content, Composition, and Localization: Influence on Muscle Insulin Sensitivity. Diabetes.

[153] Perreault L., Newsom S.A., Strauss A., Kerege A., Kahn D.E., Harrison K.A., Snell-Bergeon J.K., Nemkov T., D’Alessandro A., Jackman M.R. (2018). Intracellular localization of diacylglycerols and sphingolipids influences insulin sensitivity and mitochondrial function in human skeletal muscle. JCI Insight.

[154] Schaffer J.E. (2003). Lipotoxicity: When tissues overeat. Curr. Opin. Lipidol..

[155] Strle K., Broussard S.R., McCusker R.H., Shen W.H., Johnson R.W., Freund G.G., Dantzer R., Kelley K.W. (2004). Proinflammatory cytokine impairment of insulin-like growth factor I-induced protein synthesis in skeletal muscle myoblasts requires ceramide. Endocrinology.

[156] Guillet C., Masgrau A., Walrand S., Boirie Y. (2012). Impaired protein metabolism: Interlinks between obesity, insulin resistance and inflammation. Obes. Rev..

[157] Beals J.W., Burd N.A., Moore D.R., van Vliet S. (2019). Obesity Alters the Muscle Protein Synthetic Response to Nutrition and Exercise. Front. Nutr..

[158] Tardif N., Salles J., Guillet C., Tordjman J., Reggio S., Landrier J.F., Giraudet C., Patrac V., Bertrand-Michel J., Migne C. (2014). Muscle ectopic fat deposition contributes to anabolic resistance in obese sarcopenic old rats through eIF2α activation. Aging Cell.

[159] Cho K.A., Kang P.B. (2015). PLIN2 inhibits insulin-induced glucose uptake in myoblasts through the activation of the NLRP3 inflammasome. Int. J. Mol. Med..

[160] Wu K.K., Cheung S.W., Cheng K.K. (2020). NLRP3 Inflammasome Activation in Adipose Tissues and Its Implications on Metabolic Diseases. Int. J. Mol. Sci..

[161] Patsouris D., Cao J.J., Vial G., Bravard A., Lefai E., Durand A., Durand C., Chauvin M.A., Laugerette F., Debard C. (2014). Insulin resistance is associated with MCP1-mediated macrophage accumulation in skeletal muscle in mice and humans. PLoS ONE.

[162] Gao R., Shi H., Chang S., Gao Y., Li X., Lv C., Yang H., Xiang H., Yang J., Xu L. (2019). The selective NLRP3-inflammasome inhibitor MCC950 reduces myocardial fibrosis and improves cardiac remodeling in a mouse model of myocardial infarction. Int. Immunopharm..

[163] Wei W., Li X.X., Xu M. (2019). Inhibition of vascular neointima hyperplasia by FGF21 associated with FGFR1/Syk/NLRP3 inflammasome pathway in diabetic mice. Atherosclerosis.

[164] Muriach M., Flores-Bellver M., Romero F.J., Barcia J.M. (2014). Diabetes and the brain: Oxidative stress, inflammation, and autophagy. Oxid. Med. Cell Longev..

[165] Zhai Y., Meng X., Ye T., Xie W., Sun G., Sun X. (2018). Inhibiting the NLRP3 Inflammasome Activation with MCC950 Ameliorates Diabetic Encephalopathy in db/db Mice. Molecules.

[166] Trotta M.C., Maisto R., Guida F., Boccella S., Luongo L., Balta C., D’Amico G., Herman H., Hermenean A., Bucolo C. (2019). The activation of retinal HCA2 receptors by systemic beta-hydroxybutyrate inhibits diabetic retinal damage through reduction of endoplasmic reticulum stress and the NLRP3 inflammasome. PLoS ONE.

[167] Shippy D.C., Wilhelm C., Viharkumar P.A., Raife T.J., Ulland T.K. (2020). β-Hydroxybutyrate inhibits inflammasome activation to attenuate Alzheimer’s disease pathology. J. Neuroinflamm..

[168] Miyauchi T., Uchida Y., Kadono K., Hirao H., Kawasoe J., Watanabe T., Ueda S., Okajima H., Terajima H., Uemoto S. (2019). Up-regulation of FOXO1 and reduced inflammation by β-hydroxybutyric acid are essential diet restriction benefits against liver injury. Proc. Natl. Acad. Sci. USA.

[169] Thomsen H.H., Rittig N., Johannsen M., Møller A.B., Jørgensen J.O., Jessen N., Møller N. (2018). Effects of 3-hydroxybutyrate and free fatty acids on muscle protein kinetics and signaling during LPS-induced inflammation in humans: Anticatabolic impact of ketone bodies. Am. J. Clin. Nutr..

